# Effects of repurposed drug candidates nitroxoline and nelfinavir as single agents or in combination with erlotinib in pancreatic cancer cells

**DOI:** 10.1186/s13046-018-0904-2

**Published:** 2018-09-21

**Authors:** Serena Veschi, Laura De Lellis, Rosalba Florio, Paola Lanuti, Alberto Massucci, Nicola Tinari, Michele De Tursi, Pierluigi di Sebastiano, Marco Marchisio, Clara Natoli, Alessandro Cama

**Affiliations:** 1 Department of Pharmacy, “G. d’Annunzio” University of Chieti-Pescara, Via dei Vestini, 66100 Chieti, Italy; 2 Unit of General Pathology, Center on Aging Sciences and Translational Medicine (CeSI-MeT), “G. d’Annunzio” University of Chieti-Pescara, Chieti, Italy; 3 Center on Aging Sciences and Translational Medicine (CeSI-MeT), “G. d’Annunzio” University of Chieti-Pescara, Chieti, Italy; 4 Department of Medicine and Aging Sciences, “G. d’Annunzio” University of Chieti-Pescara, Chieti, Italy; 5 Department of Medical, Oral and Biotechnological Sciences, Center on Aging Sciences and Translational Medicine (CeSI-MeT), “G. d’Annunzio” University of Chieti-Pescara, Chieti, Italy; 6 Department of Medical, Oral and Biotechnological Sciences, “G. d’Annunzio” University of Chieti-Pescara, Chieti, Italy; 7 Division of Surgical Oncology, ASL-2 Abruzzo, “SS Annunziata” Hospital, Chieti, Italy

**Keywords:** Pancreatic ductal adenocarcinoma, PDAC, Drug repositioning, Drug combinations, Colony formation, Combination index

## Abstract

**Background:**

Pancreatic cancer (PC) is the fourth most common cause of cancer death. Combination therapies with classical chemotherapeutic agents improved treatment of advanced PC at the cost of a relevant toxicity, but the 5-year survival rate remains below 5%. Consequently, new therapeutic options for this disease are urgently needed. In this study, we explored the effect of two repurposed drug candidates nelfinavir and nitroxoline, approved for non-anticancer human use, in PC cell lines. Nelfinavir and nitroxoline were tested as single agents, or in combinations with or without erlotinib, a targeted drug approved for PC treatment.

**Methods:**

The effects of the drugs on the viability of AsPC-1, Capan-2 and BxPC-3 PC cell lines were assessed by MTT. The impact of the treatments on cell cycle distribution and apoptosis was analyzed by flow cytometry. The effects of treatments on proteins relevant in cell cycle regulation and apoptosis were evaluated by western blot. Self-renewal capacity of PC cell lines after drug treatments was assessed using a clonogenic assay.

**Results:**

When used as single agents, nelfinavir and nitroxoline decreased viability, affected cell cycle and reduced the expression of relevant cell cycle proteins. The effects on apoptosis were variable among PC cell lines. Moreover, these agents drastically impaired clonogenic activity of the three PC cell lines. Combinations of nelfinavir and nitroxoline, with or without erlotinib, resulted in dose- and cell-dependent synergistic effects on cell viability. These effects were paralleled by cell cycle alterations and more consistent apoptosis induction as compared to single agents. Treatments with drug combinations induced drastic impairment of clonogenic activity in the three cell lines.

**Conclusions:**

This study shows that two non-antitumor drugs, nelfinavir and nitroxoline, as single agents or in combination have antitumor effects that appear comparable, or in some case more pronounced than those of erlotinib in three PC cell lines. Our results support repurposing of these approved drugs as single agents or in combination for PC treatment.

**Electronic supplementary material:**

The online version of this article (10.1186/s13046-018-0904-2) contains supplementary material, which is available to authorized users.

## Background

Pancreatic cancer (PC) remains the fourth most common cause of cancer death due to poor survival rate and rapid fatality after diagnosis [1]. PC is typically diagnosed at advanced stages when the only available treatments are palliative. The poor clinical outcome of PC is attributable to early local spread, high trend of distant metastasis, resistance to traditional radiotherapy and to most systemic chemotherapies [2]. During the last decade, the overall survival of patients with advanced disease lingered around 6 months [1]. Combination chemotherapies using gemcitabine plus albumin-bound paclitaxel (nab-paclitaxel) or FOLFIRINOX (5-FU, leucovorin, irinotecan and oxaliplatin) are more effective than single chemotherapeutic agents providing a clear improvement in the treatment of PC patients with good performance status [2]. Nevertheless, each of these agents has a relevant toxicity that becomes even more marked when they are used in combination [1]. Therefore, considering their heavy side effects, only selected patients with advanced disease can tolerate such combination chemotherapies [2]. Hence, there is an urgent need to find more effective and less toxic therapeutic approaches to treat this lethal disease.

Ideally, combination therapies should include non-toxic drugs that act synergistically to kill tumor cells in a selective way. Several natural or synthetic compounds have been explored as single agents or in combination with standard chemotherapy in preclinical models of PC [3–7]. Current clinical trials are investigating the use of targeted agents in PC, which may result less toxic than conventional chemotherapy [1]. Overexpression of EGFR is reported in up to 90% of PCs [8] and several trials involved the inhibition of EGFR-MEK pathway [2]. Among the targeted agents that have been tested, erlotinib, a small molecule EGFR-tyrosine kinase inhibitor [9], was approved for the treatment of advanced PC. Erlotinib is a useful targeted agent that has been combined with other classical chemotherapeutic drugs [9].

There is a growing body of evidence that a number of non-anticancer drugs already approved for disparate human diseases have anticancer properties [10, 11]. These agents could be repurposed in cancer therapy and because of their low toxicity they could be conveniently combined, likely with less adverse effects as compared to combinations of classical chemotherapeutic agents. In addition to their low toxicity, the fact that these drugs are already approved for human use should facilitate a more rapid translation of experimental results in human therapy. Some non-anticancer drugs have been tested in vitro and in patients with PC [12, 13], but studies in this field are rather limited.

In this study, we evaluated the effects of two non-anticancer drugs, nelfinavir and nitroxoline, as single agents and/or in combinations with or without erlotinib, in PC cell lines. The three drugs are already approved for human use, have a relatively low toxicity and their combination has not been tested before. Nelfinavir is a competitive inhibitor of HIV aspartyl protease used in combination with other antiretroviral drugs to treat patients with HIV infection [14]. Chemoradiotherapy combined with nelfinavir has been tested in locally advanced inoperable PC patients, enhancing radiosensitivity with low toxicity [15, 16]. Nitroxoline is an antibiotic used for the treatment of urinary tract infections and it has been shown to affect viability and growth of different types of cancer [12, 17–19]. In the present study, we show that nelfinavir and nitroxoline, when used as single agents, significantly decrease viability, affect cell cycle, induce apoptosis and hamper clonogenicity in a cell-dependent way in the PC cell lines tested. Furthermore, their combinations with or without erlotinib produce enhanced antitumor effects, as compared to single agents.

## Methods

### Reagents and antibodies

3-(4,5-Dimethyl-2-thiazolyl)-2,5-diphenyl-2H-tetrazolium bromide (MTT), crystal violet, RNAse, propidium iodide (PI), RIPA Buffer (150 mM NaCl, 1.0% IGEPAL® CA-630, 0.5% sodium deoxycholate, 0.1% SDS, 50 mM Tris, pH 8.0) containing 1 mM PMSF and protease inhibitor cocktail were obtained from Sigma (St. Louis, MO, USA). Mouse monoclonal anti-cyclin D3 antibody and rabbit polyclonal PARP antibody were purchased from Cell Signaling Technology, Inc. (Beverly, MA, USA). Goat anti-rabbit IgG-HRP, goat anti-mouse IgG-HRP, anti-cyclin B1 antibodies were obtained from Santa Cruz Biotechnology, Inc. (Dallas, TX, USA). Monoclonal anti-β-actin antibody was obtained from Sigma (St. Louis, MO, USA).

### Cell lines and treatments

Human pancreatic cancer (PC) cell lines AsPC-1 and Capan-2 were purchased from Cell Lines Service (CLS, Eppelheim, Germany). PC cell line BxPC-3 was purchased from American Type Culture Collection (ATCC; Manassas, VA, USA). The three cell lines are known to express the EGFR protein [20, 21]. AsPC-1 and Capan-2 carry *KRAS* mutations, while BxPC-3 and AsPC-1 are *TP53* mutated. Cells were cultured in RPMI 1640, supplemented with 10% FBS at 37 °C, 5% CO_2_. Nelfinavir mesylate and erlotinib HCl (OSI-744) were obtained from Selleckchem (Munich, Germany). 8-hydroxy-5-nitroquinoline (nitroxoline) was purchased from Sigma (St. Louis, MO, USA).

### Cell viability assay

Cell viability was tested by MTT assay (Sigma, St. Louis, MO, USA). Briefly, cells were seeded in 96-well plates (4 × 10^3^ cells/well) and were treated the following day for 48 h with erlotinib, nelfinavir or nitroxoline as single agents, or with combinations of the drugs at various concentrations as indicated. Then, the MTT solution was added to each well and incubated at 37 °C for at least 3 h, until a purple precipitate was visible. In order to dissolve formazan crystals, the culture medium was replaced with dimethyl sulfoxide (DMSO, Euroclone). Absorbance of each well was quantified at 540 and 690 nm, using a Synergy H1 microplate reader (BioTek Instruments Inc., Winooski, VT, USA).

### IC_50_ and combination index calculation

IC_50_ values were calculated using the CompuSyn software. Interactions among erlotinib, nelfinavir and nitroxoline were quantified by determining the combination index (CI). The CI was calculated by CompuSyn software, based on the Chou-Talalay equation [22]: a CI < 1 indicates synergistic effects, a CI = 1 indicates additive effects and a CI > 1 indicates antagonistic effects.

### Cell cycle analysis

Approximately 0.5 × 10^6^ cells per experimental condition were harvested, fixed in 70% cold ethanol and kept at 4 °C overnight. Cells were then resuspended in 50 μg/ml PI (Sigma, St. Louis, MO, USA) and 200 μg/ml RNAse (Sigma, St. Louis, MO, USA) as previously described [23]. Cell cycle analysis (10^5^cells) was performed using a FACScanto II flow cytometer (BD, Becton-Dickinson Biosciences, San Jose, CA). Data were analyzed with FlowJo software v8.8.6 (TreeStar, Ashland, OR) and FCS Express 5 Software (De Novo Software, Glendale, CA).

### Western blot analysis

Cells were collected and lysed in RIPA buffer supplemented with protease inhibitor cocktail. Protein concentrations were determined by the BCA Protein Assay (Thermo Scientific, Rockford, IL, USA) and 30 μg were subjected to electrophoresis followed by immunoblotting. The membranes were blocked in 5% nonfat dry milk for one hour at room temperature and incubated with the appropriate primary antibodies. Then the membranes were incubated with either anti-rabbit or anti-mouse HRP-conjugated secondary antibodies. The blots were revealed by chemiluminescence using the SuperSignal West Pico Chemiluminescence Substrate (Thermo Scientific, Rockford, IL, USA) according to the manufacturer’s instructions. β-actin was used as loading control.

### Apoptosis assay

To assess apoptosis, BD Pharmingen™ APC Annexin V and 7-AAD viability staining solutions (BD, Becton-Dickinson Biosciences, San Jose, CA) were used according to the manufacturer’s instructions. Cells were resuspended in a specific binding buffer and stained using 7-AAD for dead cell exclusion and Annexin V antibody for 30 min at 4 °C in the dark. Subsequently, the samples were washed and analyzed on a FACScanto II flow cytometer (BD, Becton-Dickinson Biosciences, San Jose, CA). For each sample, at least 10^5^ events were collected. Viable cells were Annexin-V^neg^, while apoptotic cells were Annexin-V^pos^.

### Clonogenic assay

Clonogenic assay was performed essentially as previously described [24]. PC cells were seeded in 6-well plates (10^3^ cells/well) and following cell attachment they were exposed for 48 h to erlotinib, nelfinavir, nitroxoline or their combinations as indicated. Then, after medium refreshment, the plates were incubated at 37 °C with 5% CO_2_, until cells in the control vehicle formed colonies consisting of at least 30 cells (3–4 days). Colonies were fixed with 70% methanol and stained with 0.5% crystal violet, then rinsed with tap water, dried and counted.

### Statistical analysis

Statistical analyses were performed using GraphPad Prism version 5.01 software (San Diego, CA). Comparisons of mean values were performed by an unpaired Student’s t-test. Multiple comparisons were performed by one-way ANOVA followed by Dunnett’s test. A *p*-value ≤0.05 was considered statistically significant.

## Results

### Erlotinib, nelfinavir and nitroxoline affect cell viability of pancreatic cancer (PC) cell lines

**Fig. 1:**
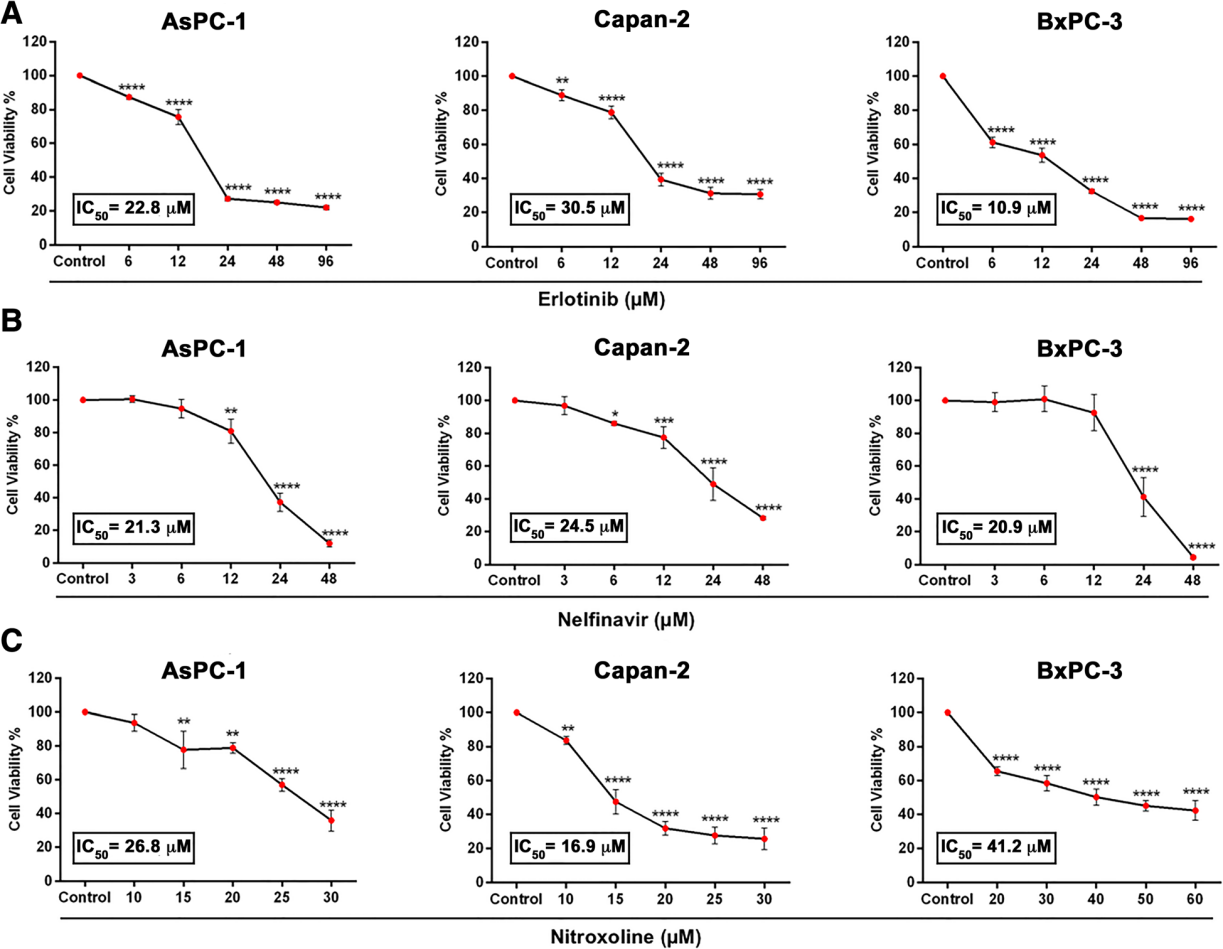
Erlotinib, nelfinavir and nitroxoline affect cell viability in AsPC-1, Capan-2 and BxPC-3. Cell viability was assessed by MTT assay after incubation for 48 h with erlotinib (**a**) nelfinavir (**b**) or nitroxoline (**c**) at the indicated concentrations, or with vehicle (control). Data shown are the means ± SD of three independent experiments with quintuplicate determinations. *Statistically significant differences between control and each drug concentration (*p < 0.05; **p < 0.01; ***p < 0.001; ****p < 0.0001)

We analyzed by MTT the effect of erlotinib, nelfinavir and nitroxoline on the viability of three PC cell lines. The three drugs significantly affected cell viability in a dose-dependent manner, with distinct sensitivities for the three cell lines (Fig. 1, panels a, b, c). In AsPC-1, nelfinavir and nitroxoline had IC_50_ values (21.3 μM and 26.8 μM, respectively) comparable to those obtained with erlotinib (22.8 μM). In Capan-2, nitroxoline had an IC_50_ value (16.9 μM) lower than those obtained with nelfinavir and erlotinib (24.5 μM and 30.5 μM, respectively). Conversely, in BxPC-3 the IC_50_ of erlotinib (10.9 μM) was lower as compared to nelfinavir and nitroxoline (20.9 μM and 41.2 μM, respectively).

### Erlotinib, nelfinavir and nitroxoline affect cell cycle in PC cell lines

**Fig. 2:**
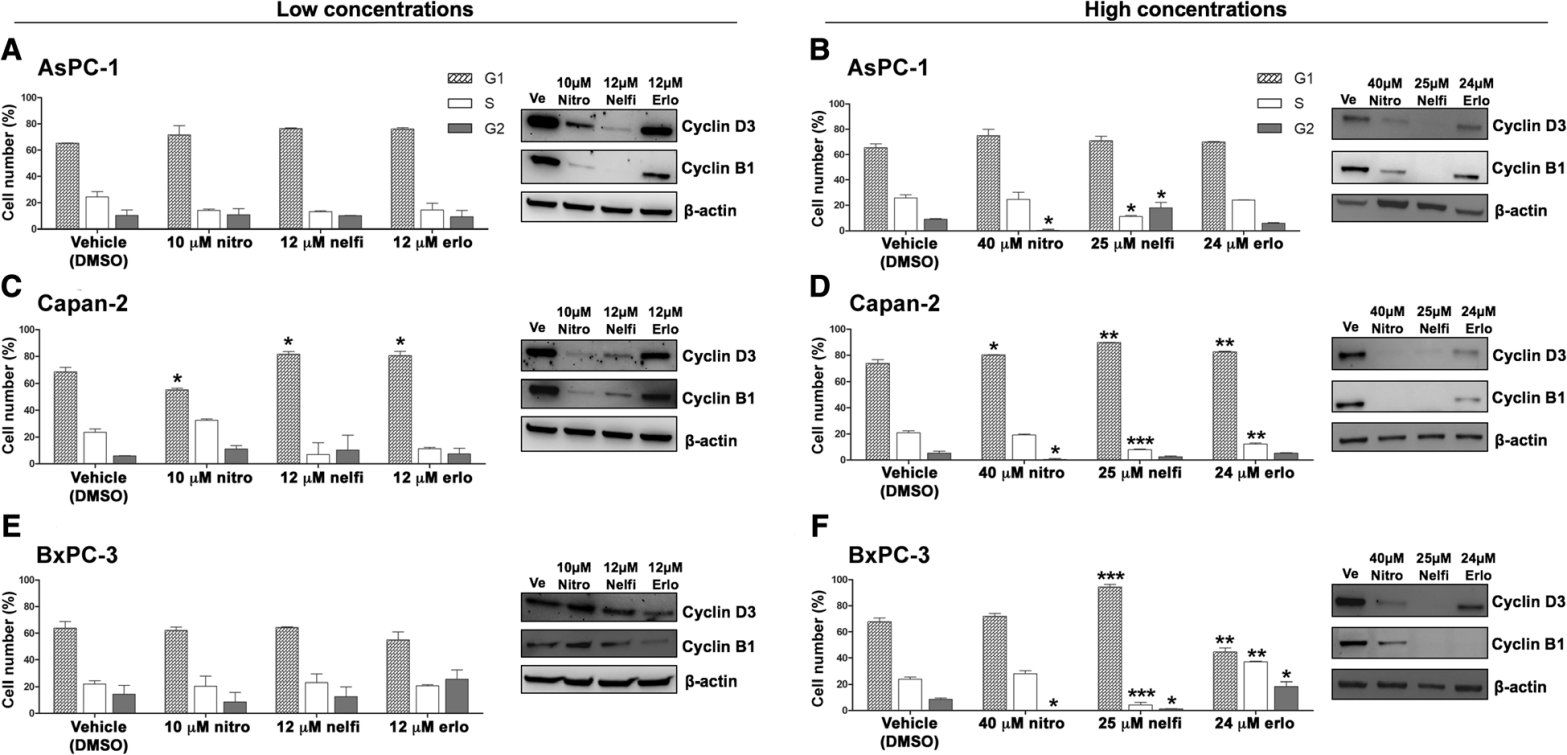
Erlotinib, nelfinavir or nitroxoline affect cell cycle in AsPC-1, Capan-2 and BxPC-3. The histograms show the mean percentage of cells in the different cell cycle phases after 72 h of treatment with vehicle, or with low concentrations (**a**, **c** and **e**, *left*), or high concentrations (**b**, **d** and **f**, *left*) of nitroxoline, nelfinavir and erlotinib as single agents. Data are the means ± SD of two independent flow cytometry experiments. The expression of cyclin D3 and cyclin B1 proteins after 48 h of treatment with the three drugs at the indicated concentrations was analyzed by western blot (A-F, *right*). *Statistically significant differences as compared to the corresponding value of vehicle (*p < 0.05; **p < 0.01; ***p < 0.001). The mean percentages of hypodiploid cells (identified as apoptotic sub-G1 population) following treatments are shown in Additional file 2: Table S2

To investigate whether the decreased PC cell viability observed in response to erlotinib, nelfinavir and nitroxoline could be due to decreased proliferation, we first evaluated cell cycle distribution by flow cytometry (Fig. 2). The PC cell lines were treated with vehicle or with two concentrations of each drug. Based on MTT results showing that the three cell lines had distinct sensitivities to each drug (Fig. 1, panels a, b, c), we selected lower concentrations of the drugs (10 μM nitroxoline, 12 μM nelfinavir and 12 μM erlotinib), which induced an approximately 20% reduction of viability, and higher concentrations of the drugs (40 μM nitroxoline, 25 μM nelfinavir and 24 μM erlotinib), which induced an approximately 50%, or greater reduction of viability.

Treatments with low concentrations of the drugs had variable effects in the three cell lines. In AsPC-1 and BxPC-3 low drug concentrations did not significantly alter cell cycle distribution (Fig. 2, panels a and e, left). In Capan-2, low concentrations of erlotinib and nelfinavir induced a G1 arrest with a statistically significant increase of the percentage of cells in this phase after treatment (erlotinib 80.55%; nelfinavir 81.60%; vehicle 68.35%), whereas nitroxoline caused a statistically significant reduction in the percentage of cells in G1 phase (55.35%) (Fig. 2, panel c, left).

Overall, high concentrations of the drugs had more evident effects across the three PC cell lines (Fig. 2, panels b, d and f, left). Erlotinib had variable effects in the three cell lines. In Capan-2, treatment with 24 μM erlotinib induced a cell cycle arrest in G1, with a statistically significant increase in the percentage of cells in this phase after treatment (82.60% vs. vehicle 73.72%) (Fig. 2, panel d, left). In BxPC-3 the same treatment induced a G2 arrest, with statistically significant increases in the percentage of cells in G2 (18.43% vs. vehicle 8.61%) and S (36.93% vs. vehicle 23.61%), with a concomitant decrease in G1 (44.65% vs. vehicle 67.78%) (Fig. 2, panel f, left). Conversely, in AsPC-1 24 μM erlotinib did not alter cell cycle distribution (Fig. 2, panel b, left). Treatment with 25 μM nelfinavir affected cell cycle distribution with distinct patterns in the three cell lines. In AsPC-1 nelfinavir induced a G2 arrest, with a statistically significant increase in the percentage of cells in this phase after treatment (17.93% vs. vehicle 8.91%) and a decrease in S phase (11.09% vs. vehicle 25.67%) (Fig. 2, panel b, left), while in both Capan-2 and BxPC-3 nelfinavir induced a G1 arrest, with a statistically significant increase in the percentage of cells in this phase after treatment (Capan-2: 89.71% vs. vehicle 73.72%; BxPC-3: 94.42% vs. vehicle 67.78%) (Fig. 2, panels d and f, left). Also treatment with 40 μM nitroxoline altered cell cycle distribution, causing a consistent reduction of the percentage of cells in G2 phase across the three PC cell lines (AsPC-1: 0.42% vs. vehicle 8.91%; Capan-2: 0.42% vs. vehicle 5.31%; BxPC-3: 0.13% vs. vehicle 8.61%) (Fig. 2, panels b, d and f, left).

To study the effects of the three drugs on the expression of relevant cell cycle proteins, we analyzed by immunoblotting the expression of cyclin D3 and cyclin B1 that are involved in G1/S and G2/M checkpoint regulation, respectively (Fig. 2). Treatments with low concentrations of the drugs had variable effects in the three PC cell lines. In BxPC-3 treatments had modest or no effects on cyclin D3 and cyclin B1 expression (Fig. 2, panel e, right), in line with the lack of cell cycle perturbation observed by flow cytometry in this cell line. In Capan-2 nelfinavir, nitroxoline and to a lesser extent erlotinib caused a reduction in the expression of cyclin D3 and cyclin B1 (Fig. 2, panel c, right), in agreement with the observation that these drugs altered cell cycle distribution by flow cytometry analysis in this cell line. Notably, in AsPC-1 nelfinavir, nitroxoline and to a lesser extent erlotinib reduced the expression of cyclin D3 and cyclin B1, but this effect did not alter the relative percentage of cells in cell cycle phases as detected by flow cytometry, suggesting a slowing down of cell cycle. At high concentrations, all treatments caused a reduction or an abolishment in the expression of cyclin D3 and cyclin B1 in the three PC cell lines (Fig. 2 panels b, d and f, right), in agreement with the observation that high concentrations of these drugs had a relevant impact on cell cycle distribution by flow cytometry. The reduction of cyclin D3 and cyclin B1 expression was less marked in AsPC-1 treated with 24 μM erlotinib, in agreement with the less evident effect of the corresponding treatment on cell cycle distribution in this cell line (Fig. 2, panel b).

Therefore, the results of flow cytometry and immunoblotting indicate that the decreased viability observed by MTT in AsPC-1, Capan-2 and BxPC-3 after treatment with erlotinib, nelfinavir and nitroxoline was at least in part related to cell cycle inhibition and that these effects were more marked and consistent at high concentrations of the drugs.

### Effects of erlotinib, nelfinavir and nitroxoline on apoptosis in PC cell lines

**Fig. 3:**
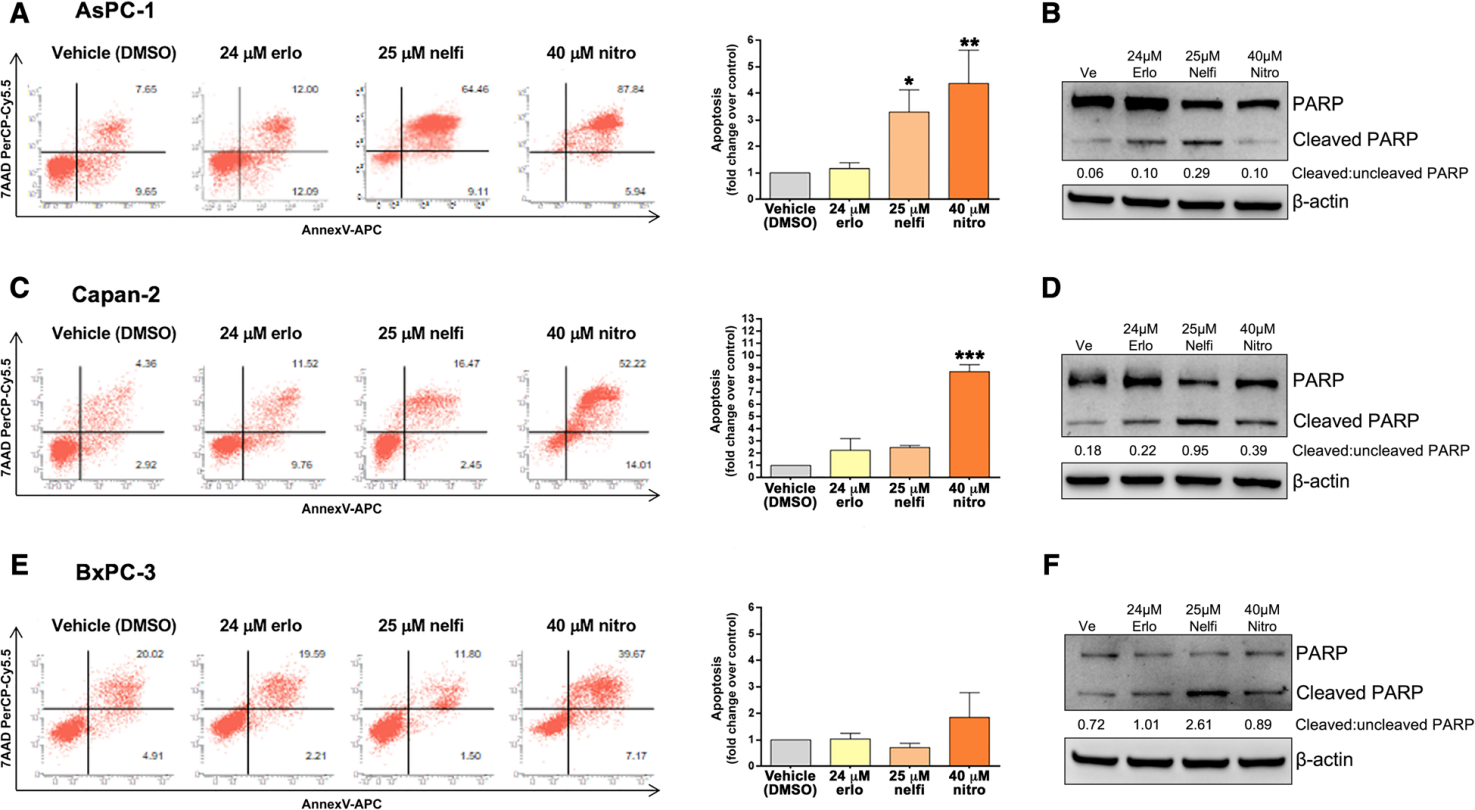
Apoptosis in AsPC-1, Capan-2 and BxPC-3 treated with erlotinib, nelfinavir or nitroxoline. Dot plots show representative experiments after 72 h of treatment with erlotinib, nelfinavir or nitroxoline in AsPC-1, Capan-2 and BxPC-3 cell lines (**a**, **c** and **e**, *left*). Values represented in the histograms are the means ± SD of at least two independent flow cytometry experiments (**a**, **c** and **e**, *right*). The expression of PARP and cleaved PARP in AsPC-1 (**b**), Capan-2 (**d**) and BxPC-3 (**f**) treated with the three drugs at the indicated concentrations for 48 h was analyzed by western blot. Ratios of cleaved:uncleaved PARP are indicated. *Statistically significant differences as compared to the vehicle (*p < 0.05; **p < 0.01; ***p < 0.001)

To evaluate whether the decreased PC cell viability observed in response to erlotinib, nelfinavir or nitroxoline could be due in part to apoptosis, we analyzed Annexin-V staining by flow cytometry (Fig. 3, panels a, c and e). Despite the use of high concentrations of the drugs, treatments did not induce consistent effects on apoptosis in the three PC cell lines. In particular, in BxPC-3 none of the drugs showed relevant effect on apoptosis by flow cytometry (Fig. 3, panel e). The effects of nelfinavir and nitroxoline on apoptosis were variable among the PC cell lines (Fig. 3, panels a, c and e). Treatment with nelfinavir resulted in a statistically significant induction of apoptosis in AsPC-1, while nitroxoline induced a marked increment of apoptotic cells in AsPC-1 and Capan-2 (Fig. 3, panels a and c) as assessed by flow cytometry. Conversely, treatment with erlotinib did not affect apoptosis in the cell lines (Fig. 3, panels a, c and e).

Also western blot analysis of PARP provided evidence of variable effects of the drugs on apoptosis in the three PC cell lines (Fig. 3, panels b, d and f). Erlotinib did not induce relevant effects on PARP cleavage as compared to vehicle across the three cell lines. Nelfinavir induced marked increases in the ratio of cleaved:uncleaved PARP as compared to vehicle in the three cell lines. Nitroxoline induced marked increases in the ratio of cleaved:uncleaved PARP as compared to vehicle only in Capan-2.

Overall, data obtained by flow cytometry and western blot analysis indicate that erlotinib did not induce relevant effects on apoptosis in the three PC cell lines. Conversely, nelfinavir and nitroxoline as single agents had variable effect on apoptosis and these were not always consistently observed in the three cell lines in the different experimental conditions used for flow cytometry and western blot analyses.

### Effects of combined treatments with erlotinib, nelfinavir and nitroxoline on PC cell viability

**Fig. 4:**
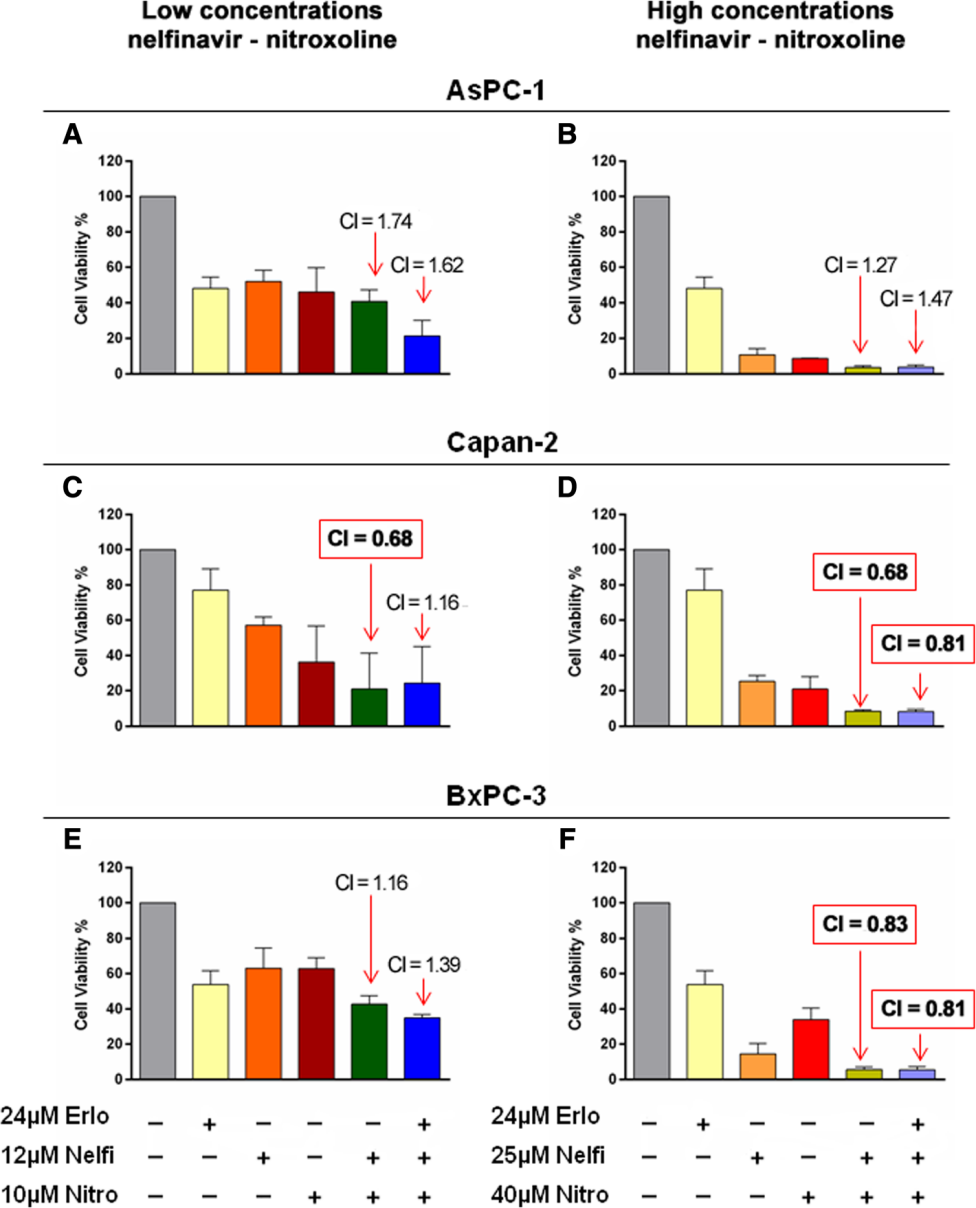
Effect of erlotinib, nelfinavir and nitroxoline as single agents or in combinations on AsPC-1, Capan-2 and BxPC-3 cell viability. Cell viability was assessed by MTT assay after incubation for 48 h with nelfinavir, or nitroxoline at low (**a**, **c** and **e**), or high concentrations (**b**, **d** and **f**) as single agents and in combinations with or without a fixed dose of erlotinib. The combination index (CI) for each drug combination was calculated by CompuSyn. To allow CI calculations two concentrations of nelfinavir, nitroxoline and erlotinib were included in each experiment. Cell viabilities at low concentration of erlotinib used for CI calculations are reported in Table S1. CIs resulted synergistic (CI < 1) for some combinations in Capan-2 (**c**, **d**) and BxPC-3 (**f**). Data shown are the means ± SD of three experiments with quintuplicate determinations

We tested the effects of combined treatments including nelfinavir and nitroxoline at lower or higher concentrations, with or without erlotinib on PC cell viability (Fig. 4).

Overall, at low and high concentrations of nelfinavir and nitroxoline the combined treatments tended to cause a greater reduction of cell viability as compared to the effect of single agents at the corresponding concentrations (Fig. 4, panels a-f and Additional file 1: Table S1). It is worth noting that the combinations of nelfinavir and nitroxoline at high concentrations without erlotinib had already a profound impact on PC cell viability and the addition of erlotinib did not increase this effect (Fig. 4, panels b, d, f and Additional file 1: Table S1).

At low concentrations, the combination between nelfinavir and nitroxoline resulted synergistic in Capan-2, as assessed by CompuSyn software (CI < 1; Fig. 4, panel c). At high concentrations, the combinations of nelfinavir and nitroxoline, with or without erlotinib, were synergistic in Capan-2 and BxPC-3 (CI < 1; Fig. 4, panels d and f). In AsPC-1, although the effects of drug combinations, especially at high concentrations, were more marked as compared to those of single agents at the corresponding concentrations (Fig. 4, panel b and Additional file 1: Table S1), combination indexes were not assessed as synergistic by CompuSyn.

In summary, these results indicate that combined treatments tended to cause a greater reduction of cell viability as compared to the effect of single agents at the corresponding concentrations, but synergistic effects were more consistently observed at high concentrations of nelfinavir and nitroxoline in Capan-2 and BxPC-3.

### Combined treatments with erlotinib, nelfinavir and nitroxoline affect cell cycle in PC cell lines

**Fig. 5:**
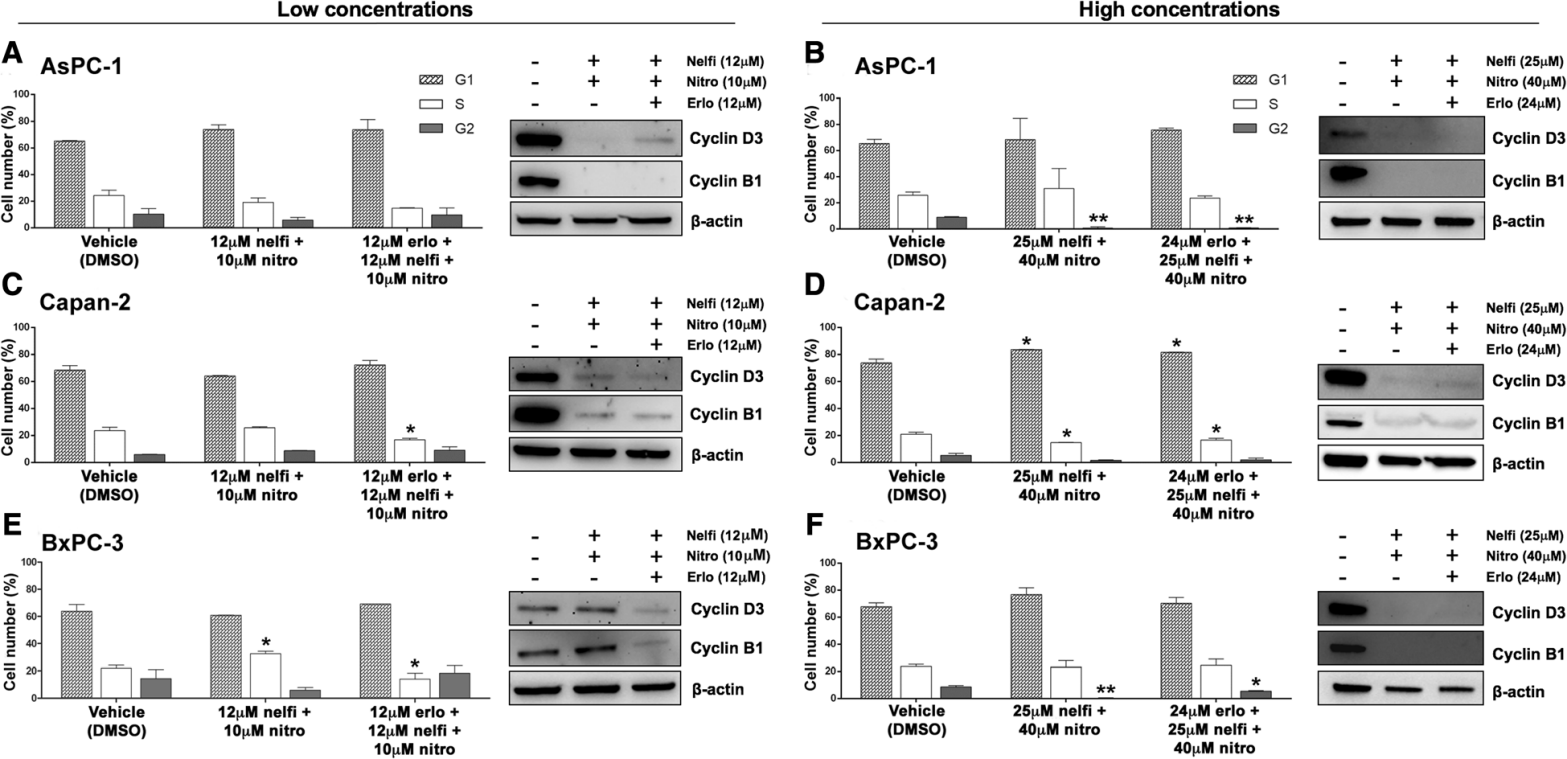
Combinations of nelfinavir and nitroxoline, with or without erlotinib, affect cell cycle in AsPC-1, Capan-2 and BxPC-3. The histograms show the mean percentage of cells in the different cell cycle phases after 72 h of treatment with low (**a**, **c** and **e**, *left*), or high concentrations (**b**, **d** and **f**, *left*) of nitroxoline and nelfinavir in combinations with or without erlotinib. Data are the means ± SD of two independent flow cytometry experiments. The expression of cyclin D3 and cyclin B1 proteins was analyzed by western blot (**a**-**f**, *right*) in cells treated for 48 h with vehicle, or drug combinations, as indicated. *Statistically significant differences as compared to the corresponding value of vehicle (*p < 0.05; **p < 0.01). The mean percentages of hypodiploid cells (identified as apoptotic sub-G1 population) following treatment are reported in the Additional file 2: Table S2

We further tested the effects of combined treatments including nelfinavir and nitroxoline at lower or higher concentrations, with or without erlotinib, on PC cell cycle distribution by flow cytometry (Fig. 5).

The combined treatment with low concentrations of nelfinavir and nitroxoline did not affect cell cycle in AsPC-1 and Capan-2 (Fig. 5, panels a and c, left), while in BxPC-3 the same combination caused a consistent increase in the percentage of cells in S phase as compared to vehicle (32.45% vs. vehicle 22.06%) (Fig. 5, panel e, left). The addition of 12 μM erlotinib to this combination produced a modest reduction in the percentage of cells in S phase in Capan-2 (16.75% vs. vehicle 23.50%) (Fig. 5, panel c, left) and BxPC-3 (14.05% vs. vehicle 22.06%) (Fig. 5, panel e, left).

The combined treatment with high concentrations of nelfinavir and nitroxoline consistently affected cell cycle in AsPC-1 and BxPC-3, causing a marked decrease in the percentage of cells in G2 phase as compared to vehicle (AsPC-1: 0.64% vs. vehicle 8.91%; BxPC-3: 0.24% vs. vehicle 8.61%) (Fig. 5, panels b and f, left). In Capan-2 this combination induced G1 arrest, with a statistically significant increase in the percentage of cells in this phase (83.66% vs. vehicle 73.72%), along with a decrease of the percentage of cells in S phase (14.73% vs. vehicle 20.97%) (Fig. 5, panel d, left). Similar patterns of alterations in the percentage of cells in the different cell cycle phases were observed across the three PC cell lines when 24 μM erlotinib was added to the drug combination (Fig. 5, panels b, d and f, left). Therefore, combined treatment with high concentrations of nelfinavir and nitroxoline affected cell cycle distribution in AsPC-1, Capan-2 and BxPC-3 with distinct sensitivities and the addition of erlotinib did not substantially modify these effects.

Western blot analysis revealed that the combination of nelfinavir and nitroxoline at low concentrations caused a marked reduction in the expression of both cyclins D3 and B1 in AsPC-1 and Capan-2, but not in BxPC-3 (Fig. 5, panels a, c and e, right). Conversely, the addition of erlotinib to this combination consistently reduced the expression of cyclins D3 and B1 across the three cell lines. Combinations of nelfinavir and nitroxoline at high concentrations, with or without erlotinib, produced a marked and consistent reduction in the expression of both cyclins D3 and B1 in the three cell lines (Fig. 5, panels b, d and f, right). Therefore, combinations of nelfinavir and nitroxoline at high concentrations, with or without erlotinib, induced a sharp decrease of cyclin D3 and cyclin B1 protein expression that was paralleled by the PC cell cycle perturbation detected using flow cytometry.

Overall, both flow cytometry and western blot analyses indicate that the decreased viability observed by MTT in AsPC-1, Capan-2 and BxPC-3 after treatments combining nelfinavir and nitroxoline, with or without erlotinib, was at least in part related to cell cycle inhibition and these effects were more marked and consistent at high concentrations of the drugs.

### Combined treatments with erlotinib, nelfinavir and nitroxoline promote apoptosis in PC cell lines

**Fig. 6:**
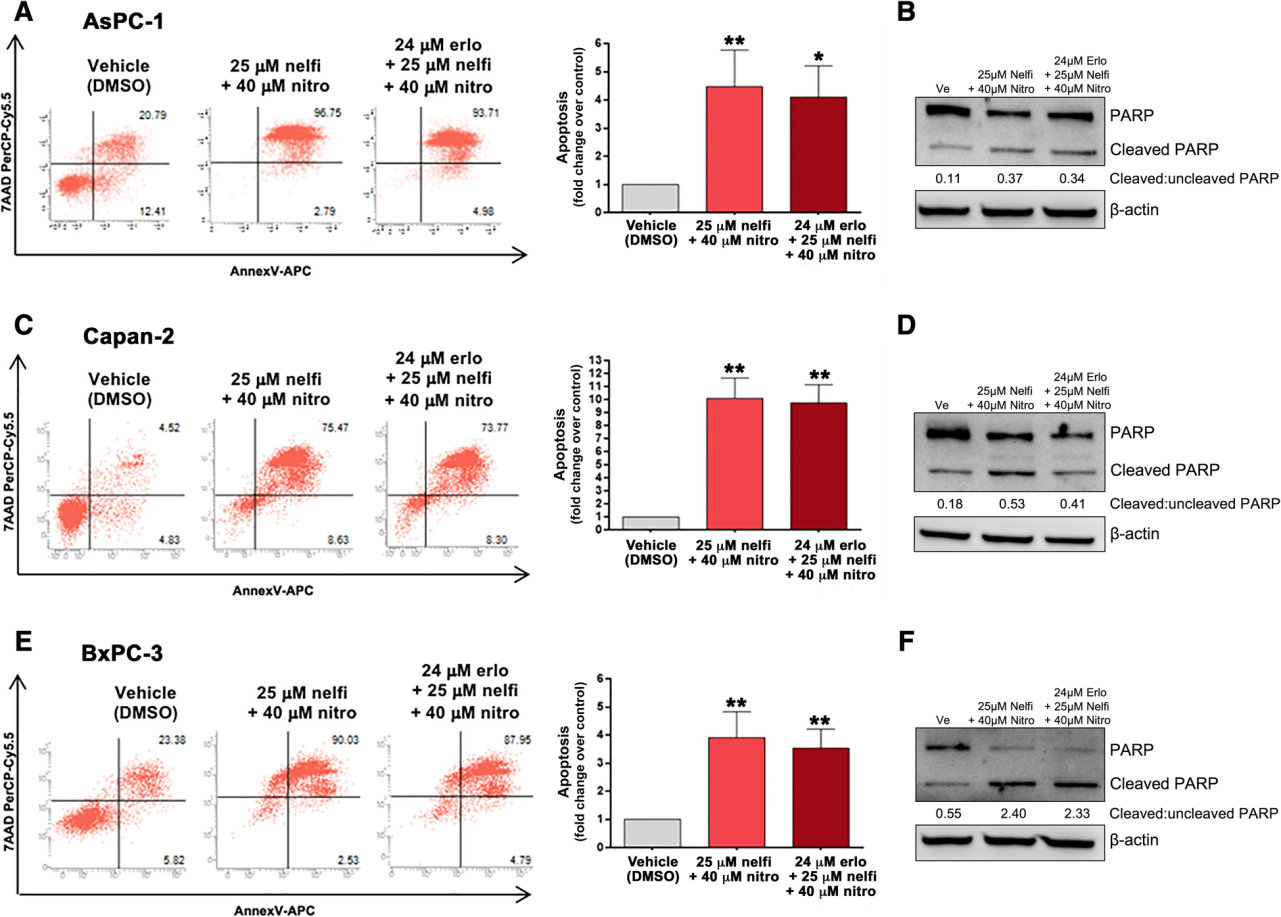
Combinations of nelfinavir and nitroxoline, with or without erlotinib, induce apoptosis in AsPC-1, Capan-2 and BxPC-3. Dot plots show representative experiments analyzing apoptosis after 72 h of treatment with combinations of nitroxoline and nelfinavir, with or without erlotinib, in PC cell lines (**a**, **c** and **e**, *left*). Values represented in the histograms are the means ± SD of at least two independent flow cytometry experiments (**a**, **c** and **e**, *right*). The expression of PARP and cleaved PARP was analyzed by western blot in AsPC-1 (**b**), Capan-2 (**d**) and BxPC-3 (**f**) treated for 48 h with combination of nitroxoline and nelfinavir, with or without erlotinib. Ratios of cleaved:uncleaved PARP are indicated. *Statistically significant differences as compared to the corresponding value of vehicle (*p < 0.05; **p < 0.01)

To analyze whether apoptosis could contribute to the marked decrease of PC cell viability observed with combined treatments (Fig. 4), we evaluated Annexin-V staining by flow cytometry (Fig. 6, panels a, c and e). The combined treatments with nelfinavir and nitroxoline, with or without erlotinib, induced a statistically significant and remarkable increment in apoptotic cells, which was consistent across the three PC cell lines (Fig. 6, panels a, c and e).

Also western blot analysis of PARP provided evidence of a marked induction of apoptosis (Fig. 6, panels b, d and f). In particular, in the three PC cell lines the combined treatments induced relevant increases in the ratio of cleaved:uncleaved PARP, as compared to vehicle. These increases were comparable in combinations with or without erlotinib, suggesting that this agent had negligible effects on apoptosis.

Overall, both flow cytometry and western blot analysis of PARP indicated that drug combinations caused a marked and consistent induction of apoptosis across the three PC cell lines.

### Effect of erlotinib, nelfinavir, nitroxoline and their combinations on PC clonogenicity

**Fig. 7:**
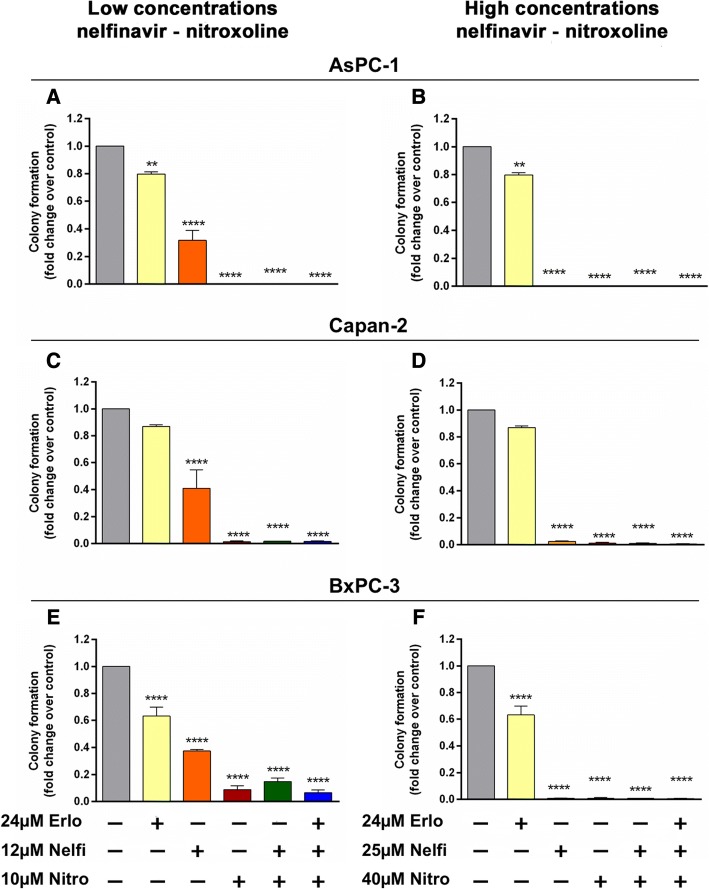
Effect of erlotinib, nelfinavir, nitroxoline and their combinations on clonogenic activity of AsPC-1, Capan-2 and BxPC-3. The effects on clonogenic activity of nelfinavir, or nitroxoline at low (**a**, **c** and **e**), or high concentrations (**b**, **d** and **f**) as single agents and in combinations, with or without erlotinib, were evaluated by a colony forming assay. Data shown in the histograms are the means ± SD of two independent experiments. Representative images of clonogenic assay are shown in Additional file 3: Figure S1. *Statistically significant differences as compared to the vehicle (**p < 0.01; ***p < 0.001; ****p < 0.0001)

We tested the effect of nelfinavir, nitroxoline and their combinations, with or without erlotinib, on clonogenicity using the same drug concentrations used for MTT assays. Treatment with erlotinib caused a modest reduction of clonogenic activity in AsPC-1, and BxPC-3 (Fig. 7, panels a, b, e, f and Table 1), while in Capan-2 no relevant effect on this activity was observed (Fig. 7, panels c, d and Table 1). Conversely, treatment with nelfinavir at low concentration caused a marked reduction of clonogenic activity across the three PC cell lines (Fig. 7, panels a, c, e and Table 1). Notably, treatment with nitroxoline at low concentration had an even greater impact, causing a drastic reduction of clonogenic activity across the three PC cell lines and the effects of this drug as single agent were comparable to those of combined treatments at low concentrations (Fig. 7, panels a, c, e and Table 1). In particular, nitroxoline and combined treatments at low concentrations substantially abolished clonogenic activity in AsPC-1 and Capan-2, whereas in BxPC-3 there was a modest residual activity either with nitroxoline, or with drug combinations (Fig. 7 panels a, c, e and Table 1). At high concentrations, except for erlotinib, all treatments with single agents or combinations substantially abolished clonogenic activity in AsPC-1, Capan-2 and BxPC-3 (Fig. 7, panels b, d, f and Table 1). Notably, high drug concentrations were extremely effective also in BxPC-3 cells that were more resistant to these treatments al low drug concentrations (Fig. 7, panels e, f and Table 1).

**Table 1:** Plating efficiency (PE) and surviving fraction (SF) values in AsPC-1, Capan-2 and BxPC-3

	AsPC-1		Capan-2		BxPC-3	
	PE %	SF %	PE %	SF %	PE %	SF %
Control	42.88	100.00	50.07	100.00	30.63	100.00
24 μM Erlo	34.28	79.94	43.50	86.88	19.37	63.22
Low concentrations of Nelfinavir and Nitroxoline						
12 μM Nelfi	14.00	32.65	20.97	41.88	11.43	37.32
10 μM Nitro	0.00	0.00	0.73	1.46	2.70	8.81
12 μM Nelfi + 10 μM Nitro	0.00	0.00	0.83	1.66	4.50	14.69
24 μM Erlo + 12 μM Nelfi + 10 μM Nitro	0.00	0.00	0.80	1.60	2.00	6.53
High concentrations of Nelfinavir and Nitroxoline						
25 μM Nelfi	0.00	0.00	1.20	2.40	0.23	0.76
40 μM Nitro	0.00	0.00	0.57	1.13	0.20	0.65
25 μM Nelfi + 40 μM Nitro	0.00	0.00	0.47	0.93	0.20	0.65
24 μM Erlo + 25 μM Nelfi + 40 μM Nitro	0.00	0.00	0.23	0.47	0.17	0.54

## Discussion

There are currently few therapeutic options for patients with pancreatic cancer (PC). Combination therapies with classical chemotherapeutic agents improved treatment of advanced PC at the cost of a relevant toxicity, but the 5-year survival rate remains below 5%. Consequently, new approaches for the treatment of this lethal disease are urgently needed. In the present study, we analyzed the effect of nitroxoline and nelfinavir, two already approved non-anticancer drugs with low toxicity. These repurposed drug candidates were tested in PC cell lines as single agents, or in different combinations with or without erlotinib, a molecule already approved for PC treatment.

The effect of nitroxoline and nelfinavir on viability was analyzed in three PC cell lines AsPC-1, Capan-2 and BxPC-3 that display different genetic profiles. Interestingly, in AsPC-1 and Capan-2 these non-toxic drugs had IC_50_ values comparable, or in some case lower than those obtained with erlotinib. Conversely, in BxPC-3 the IC_50_ of erlotinib was lower as compared to repurposed drugs. Indeed, the IC_50_ of erlotinib in BxPC-3 was also lower than the IC_50_ observed with the same drug in AsPC-1 and Capan-2. These different effects of the drugs on the viability of the three PC cell lines were possibly related to their different genetic background. It has been previously shown that nitroxoline and nelfinavir affected cell cycle in different cancer cell lines [18, 25–27]. Also the present study showed that the reduced viability observed in PC cell lines after treatment with erlotinib, nitroxoline and nelfinavir as single agents was related to an interference with cell cycle progression. The effects on the different phases of cell cycle in response to single agents were distinct in the three PC cell lines and were more consistent at high concentrations of the drugs. In particular, differently from treatments at low concentrations most treatments with single agents at high concentrations induced a cell cycle perturbation paralleled by a down-regulation in the expression of cell cycle regulators cyclin D3 and cyclin B1, which was more consistent with nitroxoline and nelfinavir in the three PC cell lines. With regard to apoptosis, its contribution to the reduced viability observed after treatment with single agents was less homogeneous. In particular, we did not observe relevant effects of erlotinib on apoptosis, whereas flow cytometry and western blot analysis provided evidence that nelfinavir and nitroxoline as single agents induced apoptosis, but these effects were not consistently observed in the three cell lines. Taken together, the above findings indicate that single agents had an impact on PC cell viability by mechanisms involving cell cycle perturbation and to a lesser extent apoptosis. As far as the effect of treatments on PC cell clonogenic ability, erlotinib showed no effect on this activity, whereas nelfinavir and nitroxoline had a drastic effect. This indicates that the two drugs candidate for repurposing have a strong impact on cell self-renewal capacity of PC cells.

Considering that there is a substantial lack of studies combining repurposed drugs in PC and that these drug combinations might have a greater antitumor effect as compared to single agents, we determined the effect of nelfinavir, nitroxoline and erlotinib combinations on PC cells. As observed with single agents, the effects were distinct in the three PC cell lines. Overall, nelfinavir and nitroxoline in combination had a more pronounced effect on viability than single agents and this effect was more consistent at high concentrations of the drugs. Notably, the addition of erlotinib to the drug combinations in most cases did not increase the effects on cell viability. At low concentrations, only the combination of nelfinavir and nitroxoline in Capan-2 was assessed as deriving from synergistic drug interactions by CompuSyn. Conversely, at high concentrations the combinations of nelfinavir and nitroxoline, with or without erlotinib, were assessed as synergistic by CompuSyn both in Capan-2 and BxPC-3. It is interesting to note that also in AsPC-1 the combinations at high drug concentrations had enhanced effects as compared to single agents, resulting in a lower residual cell viability as compared to Capan-2 and BxPC-3 with the same drug combinations. Nevertheless, in AsPC-1 drug interactions were not assessed synergistic by CompuSyn analyses. Flow cytometry and immunoblot analyses showed that the reduced viability observed in PC cell lines with drug combinations was related to an interference with cell cycle progression and was associated to a reduction of cyclin D3 and cyclin B1 protein expression, which was more marked and homogeneous across the three cell lines at high drug concentrations. The results of flow cytometry and immunoblot analyses indicated also that apoptosis contributed to the reduced viability observed after treatment with the drug combinations. In particular, the effects of drug combinations on apoptosis across the three PC cell lines were more consistent than the effects observed with single drugs, while the addition of erlotinib to the combinations did not substantially affect apoptosis. Drug combinations had also drastic effects on clonogenic ability, indicating that these combinations had a strong impact on cell self-renewal capacity in PC cell, although it should be noted that also nitroxoline as single agent had comparable strong effects. Overall, the impact of nitroxoline as single agent on clonogenicity, as well as the corresponding effect of drug combinations was more pronounced than the effects of the same treatments on viability, as assessed by MTT.

An interesting issue that is currently being investigated is how repurposed drugs exert their anticancer actions. Disparate off-target anticancer effects have been proposed and new targets are emerging for each drug. In particular, nitroxoline was shown to inhibit MetAP2, SIRT1, SIRT2 and was also shown to bind and inhibit cathepsin B, although the relevance of the latter target has been questioned because of the relatively high concentrations of the drug necessary to induce this effect [10]. Nelfinavir has been proposed to exert anticancer activity through multiple pathways including inhibition of the chymotrypsin- and trypsin-like activities of 20S human proteasome, inhibition of AKT, of hypoxia-inducible factor 1α (HIF-1α) and of HSP90, although the precise mechanism for its anticancer activity remains elusive [10]. The relative importance of these targets for the anticancer action of the two repurposed drug candidates is debated [10] and further studies will be needed to clarify this issue. Another level of complexity concerns the specific mechanism of synergistic drug combinations. It is known that drugs may simply enhance the action of other agents used in combination, or they may act as a new drug to exert effects that are distinct from individual compounds [22]. Also this issue may complicate the identification of relevant anticancer targets affected by drug combinations and will have to be investigated in future studies.

## Conclusions

Our results indicate a remarkable antitumor activity of nelfinavir and nitroxoline in PC cells. Nelfinavir and nitroxoline when used as single agents decreased viability, induced apoptosis, affected the expression of relevant cell cycle proteins, drastically compromised clonogenic activity with distinct effects in the three PC cell lines tested. These effects were enhanced by combined treatments with nelfinavir and nitroxoline. To our knowledge, this is the first study providing evidence that these human approved, non-anticancer drugs, as single agents or in combination, affect several crucial biological processes in different PC cell lines. In the experimental conditions tested in the present study the antitumor activity of these repurposed drugs appears comparable or superior to erlotinib, a targeted agent approved for PC treatment. Therefore, the results obtained with nelfinavir and nitroxoline in PC cell lines suggest that these drugs could be effectively repurposed, as single agents or in combination, for the treatment of the poorly responsive pancreatic cancer.

## Additional files

Additional file 1: **Table S1.** Effects of erlotinib, nelfinavir and nitroxoline as single agents versus drug combinations on cell viability. (XLSX 12 kb)

Additional file 2: **Table S2.** Mean percentages of cells identified as sub-G1 population. (XLSX 11 kb)

Additional file 3: **Figure S1.** Representative images of clonogenic assays. (TIF 13253 kb)

## Abbreviations

7-AAD: 7-Aminoactinomycin D
ANOVA: Analysis of variance
ATCC: American type culture collection
BCA: Bicinchoninic acid
CI: Combination index
DMSO: Dimethyl sulfoxide
EGFR: Epidermal growth factor receptor
ERLO: Erlotinib
FBS: Fetal bovine serum
HCl: Hydrochloric acid
HIV: Human immunodeficiency virus
HRP: Horseradish peroxidase
IC_50_: Half maximal inhibitory concentration
*KRAS*: Kirsten ras oncogene homolog
MEK: Mitogen-activated protein kinase kinase
MTT: 3-(4,5-Dimethyl-2-thiazolyl)-2,5-diphenyl-2H-tetrazolium bromide
NELFI: Nelfinavir
NITRO: Nitroxoline
PBS: Phosphate-buffered saline
PC: Pancreatic cancer
PE: Plating efficiency
PI: Propidium iodide
PMSF: Phenylmethanesulfonyl fluoride
RIPA: Radioimmunoprecipitation assay
RPMI: Roswell Park Memorial Institute medium
SF: Surviving fraction
*TP53*: Tumor protein p53
