# Supplementary figures and images for: Effects of repurposed drug candidates nitroxoline and nelfinavir as single agents or in combination with erlotinib in pancreatic cancer cells
Source: J Exp Clin Cancer Res. 2018 Sep 21;37:236. doi: 10.1186/s13046-018-0904-2 (PMC6151049; doi:10.1186/s13046-018-0904-2)

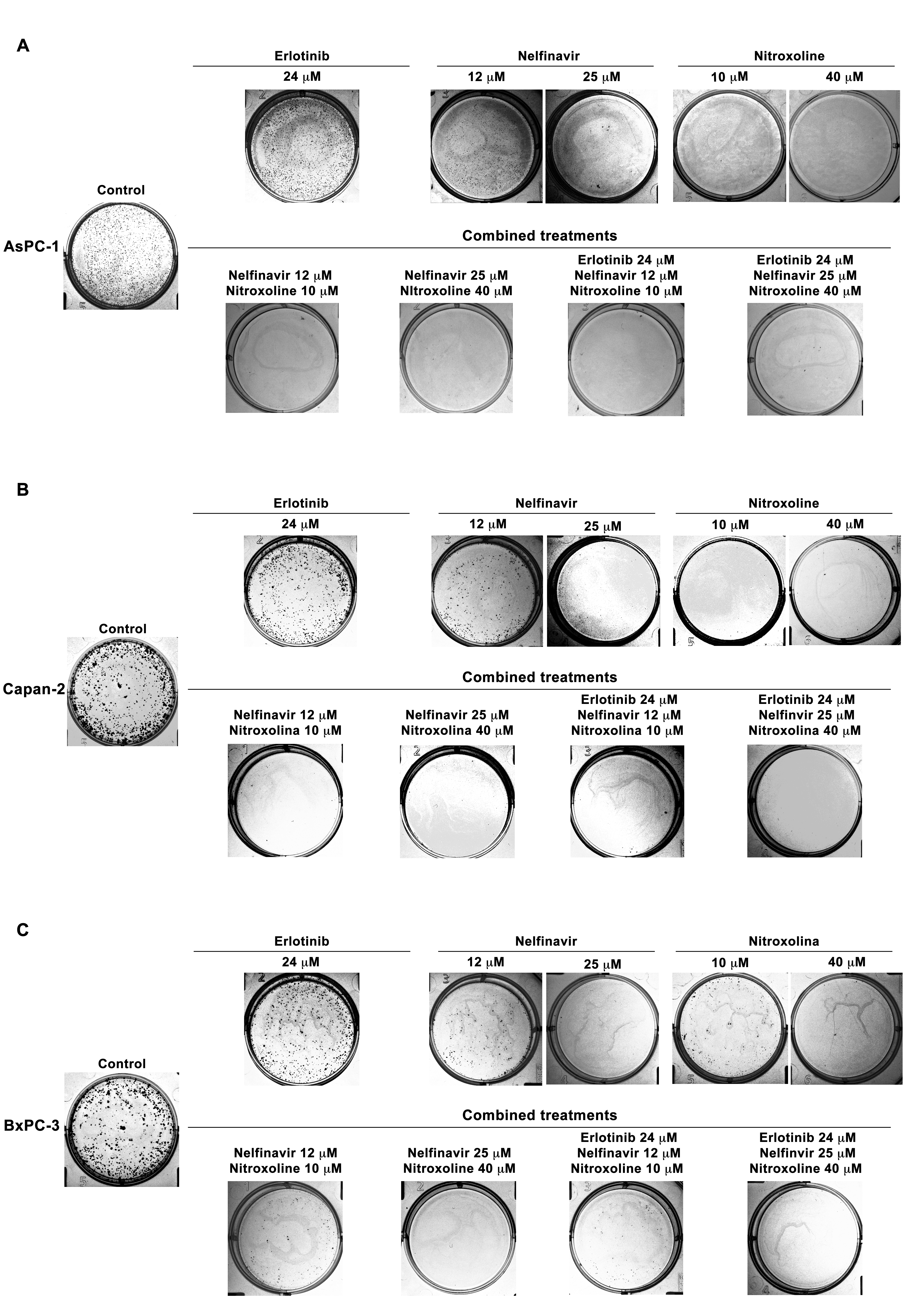

Supplement: Supplementary file 3 — Figure S1. Representative images of clonogenic assays. (TIF 13253 kb) [file 13046_2018_904_MOESM3_ESM.tif]
